# Topography-Mediated Enhancement of Nonviral Gene Delivery in Stem Cells

**DOI:** 10.3390/pharmaceutics14051096

**Published:** 2022-05-20

**Authors:** Lu Ge, Liangliang Yang, Reinier Bron, Patrick van Rijn

**Affiliations:** 1 School of Pharmaceutical Sciences, Wenzhou Medical University, Wenzhou 325035, China; gelu100466@126.com (L.G.); liangliangyanglll@126.com (L.Y.); 2 Department of Biomedical Engineering-FB40, University of Groningen, University Medical Center Groningen, 9713 Groningen, The Netherlands; r.bron@umcg.nl; 3 W.J. Kolff Institute for Biomedical Engineering and Materials Science-FB41, University of Groningen, University Medical Center Groningen, 9713 Groningen, The Netherlands

**Keywords:** topography, proliferation, gene delivery, endocytosis, transfection

## Abstract

Gene delivery holds great promise for bioengineering, biomedical applications, biosensors, diagnoses, and gene therapy. In particular, the influence of topography on gene delivery is considered to be an attractive approach due to low toxicity and localized delivery properties. Even though many gene vectors and transfection systems have been developed to enhance transfection potential and combining it with other forms of stimulations could even further enhance it. Topography is an interesting surface property that has been shown to stimulate differentiation, migration, cell morphology, and cell mechanics. Therefore, it is envisioned that topography might also be able to stimulate transfection. In this study, we tested the hypothesis “topography is able to regulate transfection efficiency”, for which we used nano- and microwave-like topographical substrates with wavelengths ranging from 500 nm to 25 µm and assessed the transfectability of human bone marrow-derived mesenchymal stem cells (hBM-MSCs) and myoblasts. For transfection, Lipofectamine 2000 and a gene encoding plasmid for red-fluorescent protein (m-Cherry) were used and topography-induced cell morphology and transfection efficiency was analyzed. As a result, topography directs cell spreading, elongation, and proliferation as well as the transfection efficiency, which were investigated but were found not to be correlated and dependent on the cell type. A 55% percent improvement of transfection efficiency was identified for hBM-MSCs grown on 2 µm wrinkles (24.3%) as compared to hBM-MSCs cultured on flat controls (15.7%). For myoblast cells, the highest gene-expression efficiency (46.1%) was observed on the 10 µm topography, which enhanced the transfection efficiency by 64% as compared to the flat control (28.1%). From a qualitative assessment, it was observed that the uptake capacity of cationic complexes of TAMRA-labeled oligodeoxynucleotides (ODNs) was not topography-dependent but that the intracellular release was faster, as indicated by the positively stained nuclei on 2 μm for hBM-MSCs and 10 μm for myoblasts. The presented results indicate that topography enhances the gene-delivery capacity and that the responses are dependent on cell type. This study demonstrates the important role of topography on cell stimulation for gene delivery as well as understanding the uptake capacity of lipoplexes and may be useful for developing advanced nonviral gene delivery strategies.

## 1. Introduction

Gene therapy is envisioned as an approach to cure diseases by delivering therapeutic genes into the cell to repair incorrect encoded genes or by introducing a new gene to modify the cell function [1,2]. In recent decades, gene delivery has gained much attention with diverse applications in biomedical sciences and applications, e.g., diagnostic devices [3], tissue regeneration [4], biosensors [5], cancer therapy [6], and vaccine development therapy [7]. Prevalently used techniques constitute: (1) Electroporation [8], microinjection (gene gun) [9], ultrasound [10], and magnetofection [11], which need expensive hardware, are time consuming, and have variable efficiency and high toxicity [12]. (2) Viral vectors, which have superior gene transfection efficiency but have limited gene packing size and insertional mutagenesis, potential immunogenicity problems, and oncogenic potential [13,14]. (3) Exploiting high-efficiency gene carriers like nonviral vectors, which attracted more and more attention due to their relative safety and simplicity of use that play a crucial role in the gene delivery process [15].

Due to the remarkable development in nanotechnology, a considerable number of excellent works have been devoted to constructing nontoxic delivery systems with the basic concepts of low toxicity and high transfection efficiency. Nonviral vehicles include cationic lipids (Lipoplexes) [15], cationic polymers (polyplexes) [16,17], organic nanoparticles [18], and inorganic nanoparticles [19]. For instance, polyethyleneimine (PEI) [20], poly-lysine (PLL), poly-amidoamine (PAMAM) [21], lipofectamine [22], and graphene quantum dots [23] are employed for systemic administration by mimicking the functions of viral cell entry that enable stronger binding affinity and avoiding the immune potential and toxicity risks of viral vectors [24]. For example, silica-based nanoparticles (SNPs), composed of spike-, hemisphere-, and bowl-type subunit nanotopographies were fabricated. The spiky surface showed the highest transfection efficacy and displayed protection against enzymatic cleavage [18]. Among them, the liposomes have gained much attention for their remarkable efficacy, simple administration, and advantageous biocompatibility to delivery macromolecules [15].

It is important to highlight that current research suggests that substrate-mediated gene delivery plays a critical role in gene delivery owing to the diverse physicochemical properties and good biocompatibility. For instance, the D. Shea group has demonstrated substrate-mediated gene transfer by immobilizing a plasmid to a cell culture substrate for the purpose of increasing the DNA concentration in the cellular microenvironment [25,26], where the adsorbed biomolecules find their way into cells for high-efficiency gene transfection. Alternatively, cells have been cultured on top of substrate and transfected via fluid transport [12]. For instance, vertical silicon nanowire interfaces [27,28,29,30] are promising vectors for the transfer of molecules because the ligand can penetrate into cell interior, which can be widely used to deliver a broad range of biological molecules (DNAs, RNAs, peptides, and proteins) into the cytosol [31]. Similarly, nanopillars of various diameters were investigated and it was reported that highest transfection efficiency of human mesenchymal stem cells (hMSCs) was induced using 200 nm diameter pillars but not nanogrooves, while transfection efficiency of monkey kidney fibroblasts (COS7) was improved on both 500 nm diameter nanopillars and 500 nm width nanogrooves by promoting the endocytosis [32]. Others found that two different types of silicon nanopillars showed different gene transfection behavior and the pillars that were broad and high achieved the highest transfection efficiency by penetrating the cell membrane while maintaining cell viability [33]. An array of aligned hollow carbon nanotubes were also created to achieve high-efficiency transfer in large quantities of cells with low cytotoxicity [12]. In addition, some studies found that nanogrooves influence gene transfection by controlling the cytoskeleton organization and nuclei morphology [2]. Exploring high-efficiency gene carriers with low toxicity is still a challenge for gene delivery.

It is known that mechanical stimuli and topography play a critical role in tissue engineering to affect cellular activities such as cell adhesion, proliferation, migration, and differentiation [34]. Our previous work demonstrates that the wavelike topography can greatly influence stem cell morphology [35], differentiation [36,37,38], and migration behavior [39] and is therefore deemed interesting for this study, not least because the found difference in behavior is substantial between the different topographies. hBM-MSCs and myoblast cells are important for tissue engineering approaches and biomedicine for their self-renewing and differentiation ability [40]. Gene delivery to mammalian cells has gained much attention in biomedical applications, diagnostic devices, tissue regeneration, biosensors, and cancer therapy but is always accompanied by low transfection efficiency [41]. Taken together, the substrate-mediated gene delivery is promising for nonviral gene transfer and the understanding of gene/carrier interactions and the mechanisms that are involved is important for successful development of a nonviral delivery system [42].

Here we hypothesize that topography is able to enhance the transfection of stem cells using standardized transfection approaches (Lipofectamine). We investigate the effect of geometrical parameters of parallel-aligned wrinkle features on transfection efficiency in hBM-MSCs and myoblast cells. To demonstrate the nonviral gene transfer, the lipid-based Lipofectamine 2000 (LF2000) reagent was used and the cellular uptake capacity of cationic complexes of TAMRA-labeled ODNs were evaluated. We also investigated the mechanical transposition of topography on cell morphology. hBM-MSCs and myoblast cells are often used in regenerative medicine and cell therapy but generally display poor transfection efficiency. We believe that using topography as the physicochemical stimulus can be presented as a low-cost and versatile culture platform that enables the understanding of topography effects on nonviral gene transfer as well as offering alternative approaches to enhance cell culture approaches.

## 2. Materials and Methods

### 2.1. PDMS Substrate Preparation

To prepare wrinkle substrates, 18 g liquid PMDS base (Sylgard 184, Dow Corning) and cross-linker was mixed in a ratio of 10:1 or 15:1. Before deposition into a cleaned squared polystyrene petri dish, the viscous mixture was degassed by applying vacuum for 15 min to remove air bubbles and was subsequently cured at 70 °C for 2 h. After curing, the elastomer slab was cut into a 9 × 9 cm substrate for further use. PDMS aligned topography substrates were prepared by stretch–oxidation–release method, as described previously [43]. To generate the aligned wave-like topography substrate, the stretching percentage, plasma oxidation parameters, and the elastomer/cross-linker ratio were varied and were summarized in Table 1.

### 2.2. Imprinting

In order to guarantee consistent chemical composition and mechanical properties and only have a difference in topography, the prepared PDMS substrates with aligned topographies served as a mold since the original prepared wrinkles vary in mechanical and chemical properties due to the different elastomer/cross-linker ratio and the oxidation times, respectively. Therefore, 30 g of fresh PDMS mixture, prepared as described above (prepolymer and cross-linker at a ratio of 10:1), was poured on top of the fabricated wrinkle mold, followed by curing at 70 °C for 2 h. After curing, the mold and fresh identical wave-like PDMS substrate were separated. Additionally, the newly prepared substrates were post-treated with air plasma at 500 mTorr for 10 min before use.

### 2.3. Topography Characterization by Atomic Force Microscopy

Topography features were characterized by an atomic force microscope (AFM) (Nano-scope V Dimension 3100 microscope, Veeco, United States) operating in tapping mode in air. Bruker SCANASYST-AIR (0.4 N m^−1^) and NP (0.017 N m^−1^) cantilevers made from silicon nitride with silicon tips (model DNP-10 tip) were used for the measurements. The wavelength and amplitude of the topographies were determined using nanoscope analysis software.

### 2.4. Cell Culture

hBM-MSCs (passage 4) were obtained from Lonza and cultured in a growth medium, including 90% Alpha modified Eagle medium (Gibco/ThermoFischer, Waltham, MA, USA), 10% fetal bovine serum (Sigma, St. Louis, MO, USA), 0.1% ascorbic acid 2-phosphate (Sigma), and 1% penicillin/streptomycin (Thermo Scientific). Cells were incubated in T75 culture flasks at 37 °C in a humidified atmosphere with 5% CO_2_. Culture medium was exchanged every 3 days. Myoblasts were kindly provided by Prof. Dr. Marco Harmsen and were the same as used in our previous studies [35,44]. The myoblasts were previously isolated from human donors undergoing reconstructive surgery with approval from the medical ethics committee. Cells displaying high self-renewal and cloning capacity that expressed cell markers Pax7, MyoD, and Myogenin were sorted by using MoFlow FACS. In this study, myoblasts (passage 8) were cultured in a growth medium containing high-glucose Dulbecco’s Modified Eagle’s Medium (Thermo/Gibco), 20% fetal bovine serum (Sigma), and 1% penicillin/streptomycin (Thermo). Cells were incubated in T25 culture flasks at 37 °C in a humidified atmosphere with 5% CO_2_. The culture medium was exchanged every 2 days and cells were harvested at the same point in time, at an almost 80% confluency. The confluent cells were routinely subcultured by Accutase (Sigma) and were used for further studies.

### 2.5. Transfection

For transfection experiments, the prepared PDMS-aligned topography with different wavelengths were cut into round shapes to fit the 24-well plate (Greiner bio-one). All the substrates were sterilized with 70% ethanol for 1 h, rinsed three times with a wash of phosphate buffered saline (PBS) to remove traces of ethanol, and then placed in 24-well plates before use. hBM-MSCs were seeded at 1 × 10^4^ cells per cm^2^ and myoblast cells were seeded at 2 × 10^4^ cells per cm^2^. At the same point, 24 h later, the cells reached 70~80% confluency, the cells were washed twice with warm Opti-MEM before the transfection procedure was used. Cationic lipid-DNA complexes (lipoplexes) were prepared as follows: 1 μL Lipofectamine 2000 and 500 ng of m-Cherry encoding plasmid were diluted in 50 μL of Opti-MEM (Thermo Fisher Scientific) separately and incubated at room temperature for 5 min and then DNA solution was added to LF2000 solution to be mixed together and incubated for 20 min. The plasmid was kindly provided by Dr. Inge S. Zuhorn and amplified from *E. coli* using GenElute HP Plasmid Mini/Midiprep kits (Sigma-Aldrich). The lipoplex solution (100 μL) was added to the cells and incubated for 4 h in a CO_2_ cell incubator. Then the cells were washed and added with a complete cell growth medium, with an additional medium change after 24 h.

Subsequently, the cells were harvested and resuspended in PBS for FACS analysis, which was performed with a BD LSR-II(λ_ex_ 488 nm/λ_em_ 530 nm) and data were further analyzed using Kaluza analysis software (Beckman coulter, Indianapolis, IN, USA). In the FSC/SSC plot, single cells were selected using a gate excluding cellular aggregation, cellular debris and small particles. A minimum of 10,000 single cells were tested. Three independent experiments were performed. The cells were then fixed by paraformaldehyde solution (4%, 30 min). After fixation, the cells were permeabilized with 1% Triton X-100 (Sigma-Aldrich Co. LLC.) and blocked with 2% bovine serum albumin (BSA, Wako Pure Chemical Industries, Ltd. sigma). Finally, the F-actin and nuclei of the transfected hBM-MSCs were stained by FITC labeled phalloidin (sigma) and 4, 6-diamidino-2-phenylin-dole (DAPI) (sigma), respectively, followed by microscopic investigation.

### 2.6. Cellular Uptake Capacity of Cationic Complexes

TAMRA-labeled oligodeoxynucleotides (ODNs) (Merck) (TAMRA-5’-AC- TACTACACTAGACTAC-3’) were applied to evaluate the cellular uptake capacity. ODNs stock solution (4 μL) and LF2000 (2 μL) were separately added into 50 μL of Opti-MEM medium for 5 min. The prepared solutions were mixed and incubated for 20 min. The cells were washed with warm Opti-MEM medium before transfection. An amount of 100 μL of transfection solution was added to each well for 30 min. To evaluate the endocytosis capacity of cationic complexes, the samples were fixed with 3.7% paraformaldehyde (Sigma) solution for 15 min. They were subsequently washed three times with PBS, then the actin filaments of hBM-MSCs and myoblast cells were stained with FITC-labeled phalloidin (Sigma, 1:200) for 1 h. The fluorescence ODNs and cytoskeleton were immediately observed using confocal microscopy (SP8X, Germany). At least 20–50 single cells were tested. Three independent experiments were performed.

### 2.7. Immunostaining

For cell morphology and the proliferation assay, hBM-MSCs and myoblast cells were seeded onto the wrinkle topographic substrates, which were cut to circular disks matching the diameter of a 24 well plate at a density of 1 × 10^4^/cm^2^ and 2 × 10^4^/cm^2^, respectively. The substrates chosen were W0.5, W2, and W25 for hBM-MSCs, and W0.5, W10, and W25 for myoblast cells. After a 24 h culture, the cells were first washed with PBS, then fixed with 3.7% paraformaldehyde (Sigma) solution in PBS for 20 min, and subsequently washed three times with PBS and permeabilized with 0.5% Triton X-100 (Sigma) solution for 3 min and blocked with 5% bovine serum albumin (Sigma) in a PBS solution for 30 min to block nonspecific binding. The cells were incubated with primary antibodies against Ki67 (Abcam, ab15580, 1:200, *v*/*v*) for 1 h. Subsequently, a secondary Texas RedX-labeled donkey anti-rabbit antibody (Jackson Immunolab, 1:100, *v*/*v*) was added for 1 h. The cell nuclei were stained by incubation with DAPI (Sigma) and cytoskeletons were stained by tetramethyl rhodamine isothiocyanate (TRITC)-phalloidin (Sigma, D9564), respectively, for 1 h. To identify proliferating cells, the percentage of ki67 positive cells was calculated at time points 12 h, 24 h, and 48 h for the chosen substrates. Finally, the cells were imaged with TissueFaxs (Tissue-Gnostics GmbH, Vienna, Austria) at 10× magnification. Cell area and ki67 positive cells were analyzed by Tissue Quest software (a high-throughput analysis technique) via fluorescent F-actin-stained cells. For each substrate, at least 400 cells were taken into consideration. Three independent experiments were performed.

### 2.8. Statistics

All data points are expressed as mean values ± standard deviation. Statistical analysis was performed with Origin 9.0 software. All data were analyzed using one way analysis of variance (ANOVA) with Tukey’s test to determine differences between groups. * *p* < 0.05, ** *p* < 0.01, and *** *p* < 0.001, respectively.

## 3. Results

### 3.1. Preparation and Characterization of Wave-like PDMS Substrates Formation

**Figure 1:**
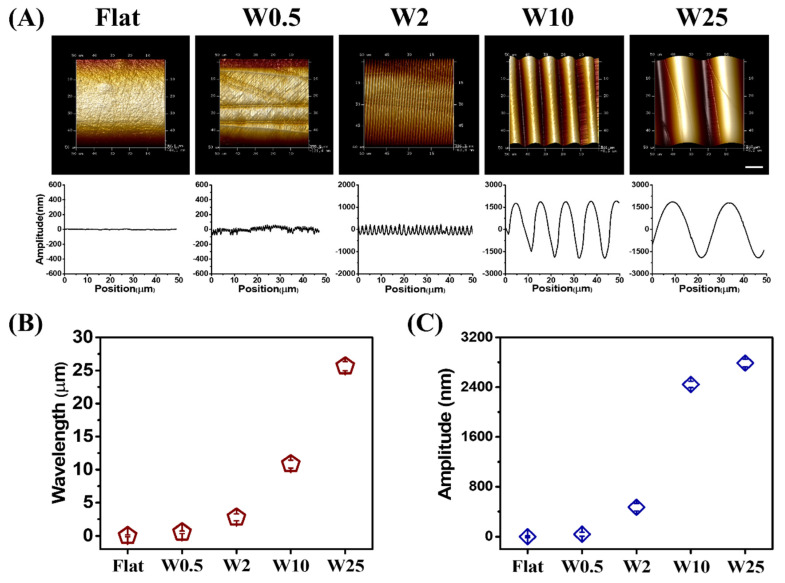
(**A**) AFM images and amplitude curves of the structured PDMS surfaces obtained after imprinting. (**B**) Wavelength and (**C**) Amplitude of created wave-like surface. Data are reported as mean ± standard deviation (SD) (*n* = 30 wrinkles). Scale bar is 10 μm and applies to all images.

The aligned wave-like topographies of PDMS-based substrates were fabricated as previously described [43]. By changing the elastomer base, pressure, stretching deformation ratio, and plasma oxidation time, the substrates were prepared with different wrinkle topography dimensions. After the preparation, an imprinting method and post-treatment with plasma oxidation was followed to guarantee the same chemical composition and mechanical surface properties with the only difference being topography. The aligned wave-like topographies were fabricated with varying wrinkle dimensions (wavelength (W; μm) and amplitude (A; μm)), which were characterized with atomic force microscopy (AFM), as shown in Figure 1A. The different surface topographies are further represented as W0.5, W2, W10, and W25, with the unstretched PDMS results on a flat surface that was used as the control. The wavelength and amplitude are dependent on one another, and both increase simultaneously, as shown in Figure 1B and Figure 1C, respectively.

### 3.2. Cell Morphology and Cytoskeletal Organization on Aligned PDMS Substrates

**Figure 2:**
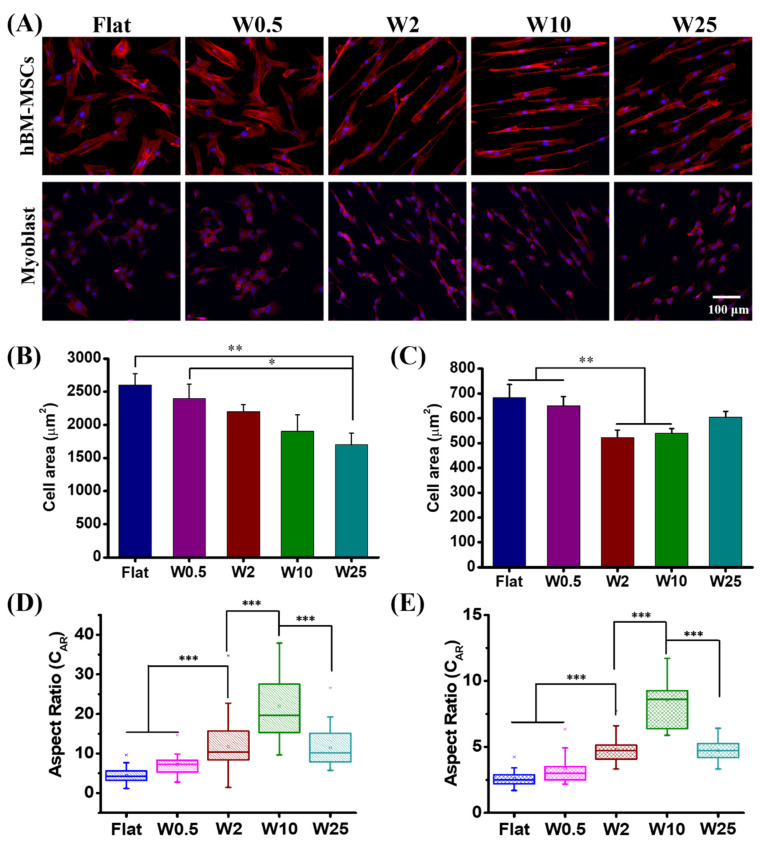
Influence of nano- and micropatterns on the morphology of hBM-MSCs and myoblast cells. (**A**) Representative fluorescence microscopy images of hBM-MSCs and myoblast cells grown on different wrinkle surfaces. F-actin and cell nucleus were stained with TRITC-labeled phalloidin (red) and by DAPI (blue), respectively. (**B**) Cell area of hBM-MSCs and (**C**) myoblast cells grown on different topographies. (**D**) Cell aspect ratio (C_AR_) of hBM-MSCs and (**E**) Myoblast cells grown on different topographies. Data are shown as mean ± standard deviation (SD), and * *p* < 0.05, ** *p* < 0.01, *** *p* < 0.001. Scale bar is 100 μm for all images.

The topographic stimuli are essential for cell function modulation and understanding the cell-material interface is a key point for tissue engineering and disease therapy [39,45]. Numerous studies indicated that cell morphology like cell spreading, alignment, and elongation is critical for cell fate decision and influences gene transfection [2,46]. In this study, parallel-aligned wrinkle features were used to examine the cell morphology change and cytoskeleton reorganization. As observed in Figure 2, after 24 h culture the cell behaviors, in terms of cell spreading and elongation, are dependent on the different substrates on which they are cultured, for both the hBM-MSCs and myoblast cells. The quantification of the cell spreading area of hBM-MSCs and myoblasts is displayed in Figure 2B,C. It can be seen that the cell area of hBM-MSCs decreased from 2500 µm^2^ to 1700 µm^2^ for those cultured on the flat control and W25, respectively. The cells grown on the flat and W0.5 display a larger cell area than on the other surfaces. In addition, the myoblast cells grown on the flat (680 µm^2^) and W0.5 (650 µm^2^) display a larger spreading area than the cells on the W2 (540 µm^2^) and W10 (560 µm^2^) topographies. The decreased cell area may be due to elongated cell morphology. The change of cell morphology on similar topographies is in line with our previous study [43].

It was found that topographic stimuli have a strong influence on cell elongation presented as the cell aspect ratio (C_AR_, defined as the ratio of the length on the major axis to the length on the minor axis of a single cell), as previously described [43]. As can be seen from Figure 2D, the C_AR_ achieved for the hBM-MSCs grown on the flat was 4.2 and the C_AR_ increased with increasing wrinkle size until W10 and then decreased when increasing the wrinkle size to W25. The C_ARS_ obtained were 7.2, 11.8, 21.9, and 11.5 for hBM-MSCs grown on W0.5, W2, W10, and W25, respectively. For the myoblast cells, the same trend is observed as for the hBM-MSCs. The cell elongation increased from the flat to W10 and then decreased again for the myoblast cells cultured on W25. The obtained C_AR_ for myoblast cells grown on flat, W0.5, W2, W10, and W25 were 2.6, 3.4, 4.8, 8.6, and 4.7, respectively (Figure 2E). These results indicate that topography has a crucial influence on cell morphology and cytoskeletal organization due to the wrinkle parameter change. However, the behavior is cell specific and while the trend for elongation is similar, the absolute elongation is different. The hBM-MSCs have a much higher tendency to elongate than the myoblast cells and the cell area is also, in general, much smaller than the hBM-MSCs and the myoblast cells do not show a continuous decrease in cell area as the hBM-MSCs do.

### 3.3. Transfection Efficiency of hBM-MSCs on Wave-like PDMS Substrates

**Figure 3:**
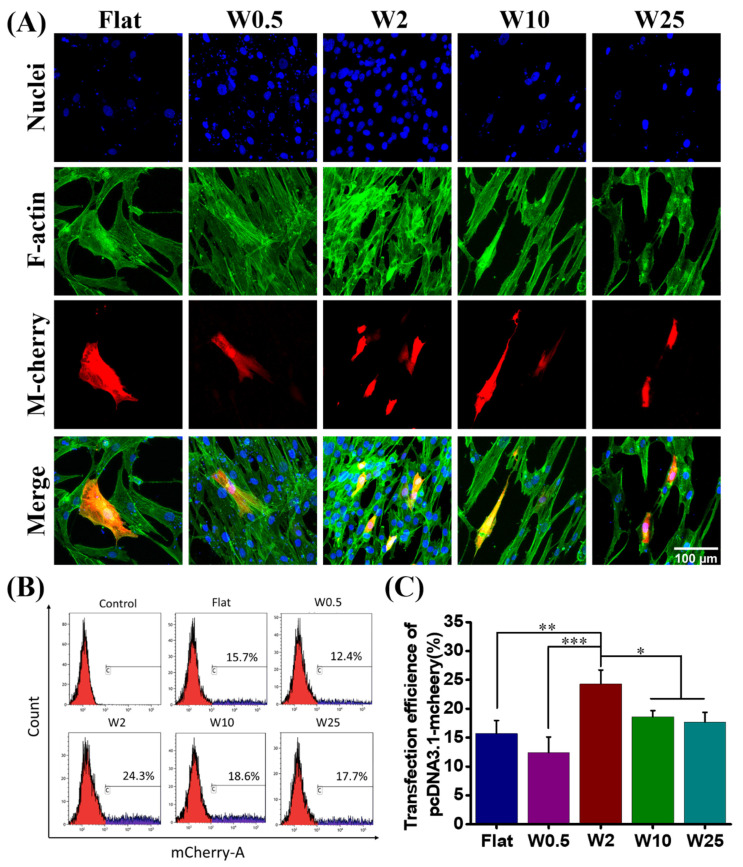
Influence of nano- and micropatterns on the transfection capacity of hBM-MSCs. (**A**) Representative fluorescence microscopy images of transfect hBM-MSCs after 24 h. F-actin and cell nucleus were stained with FITC-labeled phalloidin (green) and by DAPI (blue), respectively. The transfected cells are m-cheery express cells (red). (**B**) Flow cytometry profiles obtained after the treatment with LF2000/pDNA encoding m-cheery proteins. The percentage of transfected cells was evaluated and hBM-MSCs cells without a transfection agent were selected as the control. (**C**) Quantification data of transfected cells grown on different topographies. Three independent experiments were presented. Data are shown as mean ± standard deviation (SD), and * *p* < 0.05, ** *p* < 0.01, *** *p* < 0.001. Scale bar is 100 μm and applies to all images.

The hBM-MSCs cells are well known for their difficult transfection by nonviral based methods [47]. To evaluate the influence of wave-like topography on the transfection efficiency, hBM-MSCs were cultured on the various nano- and micropatterns for 24 h. After incubation, these cells were transfected with plasmid-encoded m-Cherry using Lipofectamine 2000 (LF2000) (Thermo fisher) as the transfection agent, according to established cell transfection protocols. The transfection was qualitatively assessed by the visualization of m-Cherry positive cells using confocal fluorescence microscopy imaging (Figure 3A). For quantitative analysis, cells were harvested by Accutase (Sigma) from the different substrates and collected for flow cytometry analysis (FACS) (Figure 3B).

We evaluated the DNA transfection capacity of hBM-MSCs on five different wrinkle topographies. The transfection behaviors of hBM-MSCs are shown in Figure 3A and the quantified data are shown in Figure 3B,C, which demonstrate that the nano- and microtopography are able to have an impact on the cell transfection efficiency. The percentage of transfected cells was evaluated by FACS and quantification results are shown in Figure 3C. The m-Cherry expression, as an indication for transfection efficiency, was lowest for cells cultured on W0.5 (12.4%), slightly lower than found on the flat (15.7%). The larger topographies had a positive effect on the transfection. The highest transfection was achieved for hBM-MSCs cultured on W2 (24.3%), while W10 (18.6%) and W25 (17.7%) displayed lower transfection efficiencies, albeit still higher than for the flat control. These results suggest that the efficiency of gene transfection can be modulated by topography, in this case, aligned wave-like structures.

### 3.4. Transfection Efficiency of Myoblast Cells on Wave-like PDMS Substrates

Undifferentiated muscle cell lines (myoblasts) are another well-known cell type that is difficult to transfect [48]. The topography-induced transfection efficiency of myoblast cells was also investigated. LF2000/DNA complexes encoding m-Cherry protein were used, similar as for the hBM-MSCs, to transfect myoblast cells. The myoblast cells were freely grown on aligned wrinkle surfaces for 24 h, after which they were transfected with LF2000/DNA complexes. The transfected cells were visualized by confocal microscopy and the percentage of m-Cherry positive cells was quantified by FACS.

**Figure 4:**
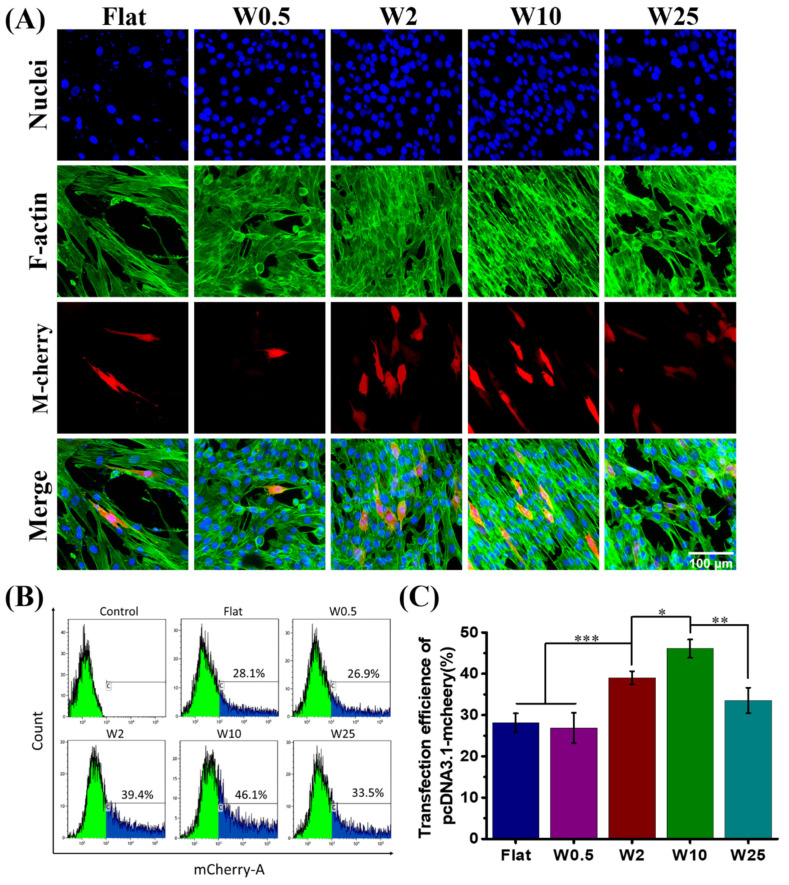
Influence of nano- and micropatterns on the transfection capacity of myoblast cells. (**A**) Representative fluorescent images of transfected myoblast cells on wave-like PDMS. F-actin and cell nuclei were stained with FITC-labeled phalloidin (green) and by DAPI (blue), respectively. Successfully transfected cells are expressed as m-Cherry (red). (**B**) Flow cytometry profiles obtained after the treatment with LF2000/p-DNA encoding m-Cherry proteins. (**C**) Quantification data of transfected cells grown on different topographies. Three independent experiments were per-formed. Data are shown as mean ± standard deviation (SD), and * *p* < 0.05, ** *p* < 0.01, *** *p* < 0.001. Scale bar is 100 μm and applies to all images.

As Figure 4A shows, the DNA was successfully transfected to the myoblast cells cultured on the winkle surface from the flat to 25 µm, but the topography has significant influence on the transfection efficiency. There are more positively transfected cells grown on W10 than the other substrates. As the FACS results show in Figure 4B, together with the quantification data shown in Figure 4C, the transfection efficiency increased on W10 with respect to the flat and then decreased on W25. Flat and W0.5 have a similar influence, while the other topographies display a positive effect on the transfection efficiency, with W10 being the most efficient in stimulating the transfection. Unlike hBM-MSCs, myoblast cells cultured on the W10 showed the highest transfection efficiency (46.1%), far more than even the highest transfection efficiency achieved of hBM-MSCs cells growth on W2 (24.3%). The percentage of m-Cherry positive cells was similar for cells cultured on W0.5 (26.9%) and the flat (28.1%). These results suggest that physical stimuli, such as topography, have an impact on the transfection efficiency, which is cell type dependent.

### 3.5. Influence of Topography on Uptake of Cationic Complexes

**Figure 5:**
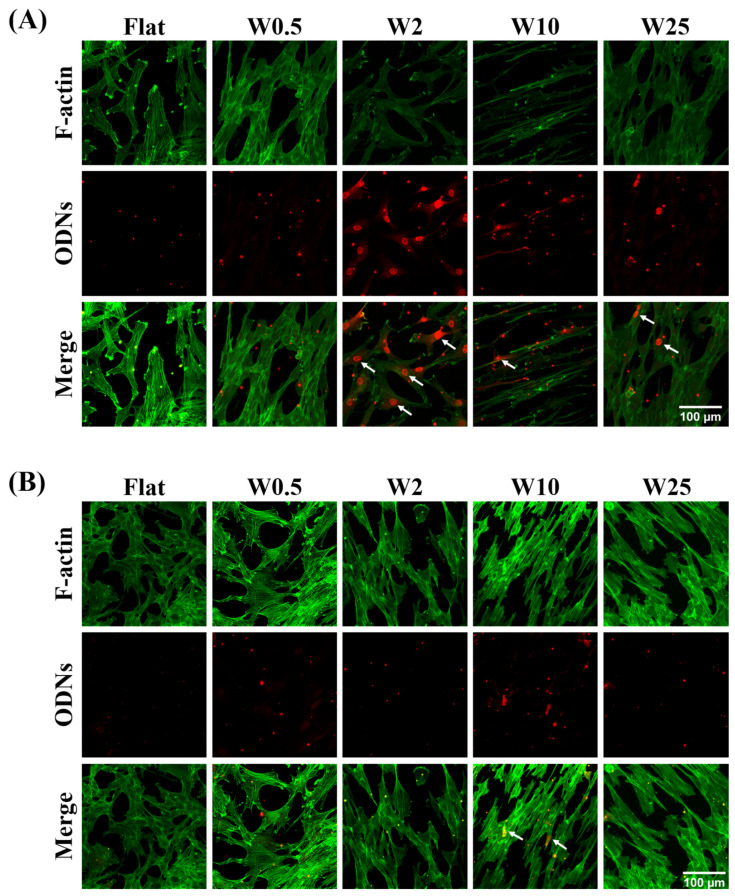
Influence of nano- and micropatterns on the cellular uptake capacity of cationic complexes. (**A**) Representative fluorescence images of hBM-MSCs cells cultured on wave-like PDMS, showing the cellular uptake of oligonucleotides (red). F-actin was stained with FITC-labeled phalloidin (green). (**B**) Representative fluorescence images of myoblast cells cultured on wave-like PDMS showing cellular uptake of oligonucleotides (red). F-actin was stained with FITC-labeled phalloidin (green). Scale bar is 100 μm and applies to all images. White arrows mark positive nuclei.

In order to gain insight into the possible origin of increased transfection, the influence of topography on the cellular uptake capacity of cationic complexes was investigated by means of Lipofectamine 2000-modified fluorescence oligodeoxynucleotides uptake studies. Cells freely grown on the wave-like topographies for 24 h were treated with the fluorescent cationic complexes by incubation for 30 min. The cells were then fixed and stained immediately. The cytoskeleton was stained with FITC-labeled phalloidin (green). The uptake of cationic complexes (red) by cells cultured on wrinkle surfaces was observed by confocal microscopy (Figure 5A). The hBM-MSCs cellular uptake capacity of ODNs was observed by the presence of endocytosed complexes shown as red dots, which is shown in Figure 5A. As the results in Figure 5A indicate, the cells grown on Flat or W0.5 substrate show a different behavior of release of the ODNs inside the cell to cells cultured on other substrates. For instance, as the white arrow indicates, cationic complexes of hBM-MSCs grown on the W2 not only penetrate the cell and appear as red dots in hBM-MSCs on Flat and W0.5 but also escape the endosome, as visualized by their nuclear accumulation. Even diffuse cytosolic staining is seen for hBM-MSCs cultured on W2. Additionally, hBM-MSCs on W10 and W25 topography display some positive nuclei, albeit less than on the W2. For the myoblast cells, as Figure 5B indicates, the topography plays a role in the uptake of cationic complexes. Even though there is no obvious nuclear localization, the enhanced cationic complex uptake capacity can be observed for myoblast cells on the W10, while the positive nuclei were not identified on the other surfaces. Taken together, the results indicate that the topography has significant effects on the gene transfection of hBM-MSCs and myoblast cells by regulating endosomal release capacity of cationic complexes. The W2 and W10 were beneficial for the endosomal escape of cationic complexes and thus transfection efficiency of hBM-MSCs and myoblast cells, respectively.

### 3.6. Influence of Topographical Structures on Cell Proliferation

**Figure 6:**
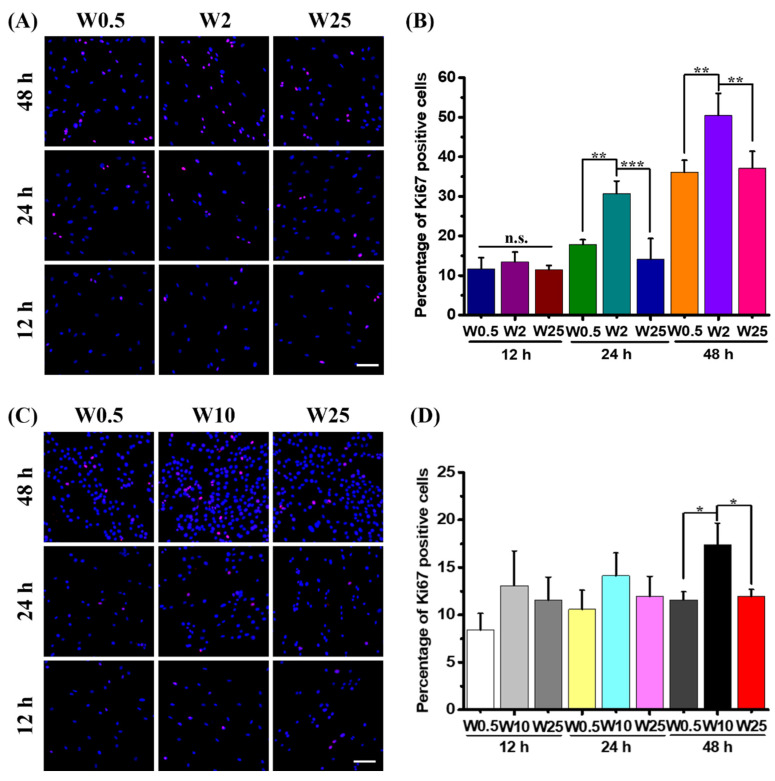
Cell proliferation assay. (**A**) Representative fluorescence images hBM-MSCs cells cultured on W0.5, W2, and W25 for 12 h, 24 h, and 48 h. The ki67 positive cells were stained in red, and cell nuclei were counterstained in blue (DAPI). (**B**) Quantification results of the percentage of Ki67 positive cells of hBM-MSCs at 12 h, 24 h, and 48 h for the chosen substrate. (**C**) Representative fluorescence images myoblast cells cultured on W0.5, W10, and W25 for 12 h, 24 h, and 48 h. (**D**) Quantification results of the percentage of Ki67 positive cells of myoblast cells at 12 h, 24 h, and 48 h for the chosen substrate. Three independent experiments were performed. Data are shown as mean ± standard deviation (SD), and * *p* < 0.05, ** *p* < 0.01, *** *p* < 0.001, n.s. no significant difference. Scale bar is 100 μm and applies to all images.

It is well established that topography plays a critical role in cellular activities, such as cell adhesion, migration, and proliferation [35]. Additionally, cell division is known to stimulate transfection with pDNA, because during mitosis the nuclear membrane is gone and the DNA can easily enter the nucleus [49]. Therefore, to explore whether the cell proliferation plays a role in gene transfection efficiency, cell proliferation assays were performed for hBM-MSCs and myoblast cells on both low-transfection and high-transfection substrates. Thus, the hBM-MSCs growth is stimulated on W0.5, W2, and W25 and myoblast cell growth is stimulated on W0.5, W10, and W25, respectively. To identify proliferating cells, the percentage of ki67 positive cells was calculated at the time points of 12 h, 24 h, and 48 h on the chosen substrates. Since hBM-MSCs and myoblast cells on W0.5 showed lower transfection efficiency than the control, as Figure 6A indicates, the percentage of ki67 positive cells depends on the substrate on which of these are cultured and the increase in cell number over time. The quantified results in Figure 6B show that there is no significant difference for ki67 positive cells of hBM-MSCs at 12 h, the percentage of ki67 positive cells is about 11.6%, 13.5%, and 11.5%, respectively. However, at 24 h, the ki67 positive cells on W2 are about 30.7%, which is more than W0.5 (17.8%) and W25 (14.1%). Additionally, the percentage of ki67 positive cells at 48 h on W2 is about 50.5%, which is significantly more than hBM-MSCs grown on W0.5 (36.1%) and W25 (37.2%).

For the myoblast cells, as Figure 6C shows, the percentage of ki67 positive cells are influenced by the substrate. There are more positive cells on W2 than on the others after 48 h. The cell number increased over time. The quantified results in Figure 6D show that there is no significant difference after a 12-h and 24-h culture in regard to the percentage of proliferating cells. The percentages of ki67 positive cells are about 8.4%, 13.1%, and 11.5% for myoblast cells on W0.5, W10, and W25 at 12 h, and 10.6%, 14.1%, and 11.9% at 24 h, respectively. However, for the ki67 positive cells of myoblast cells at 48 h, the percentage on W10 is about 17.4%, which is significantly more than the cells on W0.5 and W25. Taken together, the topography has an influence on cell proliferation, and the increased cell proliferation may be correlated with higher transfection efficiency.

## 4. Discussion

In recent years, considerable research has been focused on nonviral gene delivery vehicles, including cationic lipids, cationic polymers, and inorganic nanoparticles [50]. Notably, substrate-mediated gene delivery enables stronger binding affinity, low toxicity, high efficiency, and avoids immune response problems or risks associated with viral vectors, which is of great importance for successful transfection [32]. Even though several studies have been devoted to constructing nontoxic delivery systems, further efforts are required to understand topography on the gene transfection of adhered cells and fabricated topographic substrates with low toxicity and high efficiency. In this study, aligned topography fabricated by a silicone stretch–oxidation–release method and imprinting lithography induces the formation of aligned topography (wave-like structures). The nano- and micropatterned PDMS substrates were used to investigate the topography effect on gene delivery in hBM-MSCs and myoblast cells.

As can be seen from the hBM-MSCs and myoblast cells grown on the prepared wrinkle surface with a wavelength from flat to W25 and subsequently transfected with lipofectamine 2000 modified plasmid encoding m-Cherry proteins, the cells cultured on different topographies showed different degrees of transfection efficiency. Unlike the normally low transfection efficiency of 10%–15% for hBM-MSCs, 24.3% transfection efficiency can be achieved on W2, as shown in Figure 3B,C. The transfection efficiency was enhanced by 55% on the W2 as compared to the flat substrate. Figure 4B,C showed the transfection efficiency of myoblast cells on W2 to be about 39.4%, and the highest percentage is about 46.1% on the W10. The transfection efficiency of myoblast cells was enhanced by 64% on the W10 as compared to the flat substrate. Taken together, the topography has a great influence on nonviral gene transfer, and the percentage of transfected cells is cell-type dependent.

Some recent work demonstrated that cell morphology influences mesenchymal stem cell transfection and well-spread and elongated morphology promotes gene transfection [46]. On the other hand, other studies established that aligned, parallel f-actin and an elongated nucleus morphology of myoblast cells can reduce gene expression by PEI-mediated gene transfection [46]. In our work, the cell spreading area was altered by topography. A larger area was achieved on the flat and W0.5 substrates (Figure 2B,C), which was associated with a lower gene transfection efficiency for both hBM-MSCs and myoblast cells. Except for the cell area, as Figure 2D,E showed, the cell elongation was also modulated by the topography. The highest elongated cell shape of hBM-MSCs was achieved on W10, which showed higher transfection efficiency than the flat and W0.5 but less than W2, which displayed less cell elongation than W10. For the myoblast cells, the most elongated cell shape was achieved on W10, which showed higher gene expression than other surfaces, which is consistent with previous work [46]. The relationship between cell area, cytoskeleton organization, and gene delivery does not provide a unified answer and appears to vary between cell types. Cell proliferation has an influence on gene transfection, because when cells dive into mitosis, the disappearance of the nuclear membrane allows the pDNA to enter the nucleus more easily. As the results showed in Figure 6A–D, the percentage of proliferated hBM-MSCs is significantly more on W2 than other surfaces at 12 h, 24 h, and 48 h. Moreover, the percentage of positively proliferated myoblast cells on W10 is more than others at 48 h. Taken together, the topography-induced cell proliferation is most likely correlated with the enhanced gene transfection efficiency.

The successful therapeutic gene delivery to the target cells requires overcoming biological barriers like the negatively charged cell membrane, endosomal escape, and cell nucleus entry [51]. Thus, the cellular uptake and endosomal escape is vital to gene transfection [52]. The influence of topography on endosomal escape capacity was investigated using fluorescently labeled oligonucleotides complexed with LF2000. Figure 5A indicates that cationic complexes were processed more by the hBM-MSCs on W2 than cells grown on flat or W0.5. hBM-MSCs on the W2 substrate showed more nuclei being positively stained and even displayed more diffuse cytosolic staining than on the other substrates. On the W10 and W25 topography, some positive nuclei were also observed, albeit not as much as on W2. Figure 5B indicated that the uptake of lipoplexes were processed by fibroblast cells grown on W10. Even though it is less pronounced, more positive nuclei can be observed on the W10 in fibroblast cells than on other substrates. The successful therapeutic genes delivered to the designated target cells require overcoming of biological barriers, for instance, the cell membrane was negatively charged and rejected the DNA anionic phosphate backbone [15,53]. The efficiency of endosomal escape plays an important role in gene delivery and there are two main pathways of endocytosis, as summarized [54]: (1) Phagocytosis involves particles being taken up by the cell through vesicles in the micrometer range incorporated into the plasma membrane, and (2) Pinocytosis involves fluids containing particles being taken up by smaller vesicles. This endocytic pathway can be divided into Macropinocytosis, which is responsible for large complexes (>0.2 μm) being engulfed into a large invagination to avoid lysosomal degradation and receptor-mediated endocytosis (RME). RME involves Clathrin-mediated endocytosis and Caveolae-mediated endocytosis. Thus, in addition to overcoming the ECM protein barriers, the cargo needs to overcome cellular barriers, such as the cell membrane, successfully escape from the endosome into the cytosol, and the trafficking can be promoted by elements of the cytoskeleton located in the nucleus, which transcript the coding gene to a therapeutic protein. Here we investigated lipoplex-mediated endosomal release, which was previously shown by the Zuhorn group to rely on lipid mixing between lipoplex and the endosomal membrane [55,56] and requires complex internalization via the cholesterol-dependent clathrin-mediated pathway of endocytosis [57]. Our previous studies have proven that the nanowave-like structure has an influence on cell mechanotransduction, possibly through focal adhesion, cytoskeletal tension, and YAP signaling pathways [36,37,58,59]. Rho GTPase activation is essential for focal adhesion assembly and disassembly, while focal adhesions anchor actin stress fibers and in turn facilitate intracellular vesicular trafficking, which may affect transfection efficiency [60]. Future endeavors may therefore focus on understanding the roles of specific mechanotransductions and endocytosis pathways, which would indicate that by means of physicochemical stimuli one can modulate these mechanisms; therefore, specific chemical modifications of the transfection complexes might not be necessary.

## 5. Conclusions

In this study, topographically patterned surfaces of variable wavelengths and amplitudes were applied in order to study the guidance of the gene transfection of hBM-MSCs and myoblast cells as well as the escape capacity of endosomes from cationic complexes. This study indicates that the topography dimension has an influence on cell spreading, elongation, proliferation, and transfection behaviors. Higher transfection efficiency can be achieved on the W2 for hBM-MSCs and W10 for myoblast cells. There was an increase in efficiency of 55% and 64% for the hBM-MSCs on W2 and myoblasts on W10, respectively, as compared to the transfection efficiency on flat substrates. The increased gene delivery ability was found to be most likely related to the enhanced release and nuclear entry of genetic cargo and cell proliferation rate. This study demonstrates that topography affects gene transfection and provides useful information on in vitro gene delivery, which has applicable therapeutic potential for nonviral gene transfer. Although one can perceive the increase in the efficiency of transfection models, in terms of patient-specific cell harvesting and subsequent transfection, all numbers count, and these models can be further enhanced in relatively simple, yet effective ways, as shown here.

## Figures and Tables

**Table 1:** Conditions for preparing aligned PDMS substrates of different sizes.

Substrate	Ratio of Prepolymer and Cross-Linker	Pressure of Plasma	Time	Stretch Percent (%)
Flat	10:1			0
W0.5/A0.05	10:1	14 Torr	60 s	30
W2/A0.5	10:1	25 mTorr	15 s	30
W10/A3.5	10:1	25 mTorr	650 s	20
W25/A3.6	15:1	25 mTorr	25 min	10

Wavelength and amplitude are abbreviated as W and A, respectively, and the unit for W and A is μm.

## Data Availability

Data can be directly obtained from the authors.
